# The Clinically Significant Changes in the Composition and Functional Diversity of the Vaginal Microbiome in Women with Type 2 Diabetes Mellitus

**DOI:** 10.3390/microorganisms13061426

**Published:** 2025-06-19

**Authors:** Min Jeong Kim, Jaeeun Yoo, Soonjib Yoo, Mi Yeon Kwon, Seungok Lee, Myungshin Kim

**Affiliations:** 1 Department of Obstetrics and Gynecology, Bucheon St. Mary’s Hospital, College of Medicine, The Catholic University of Korea, Seoul 06591, Republic of Korea; poouh74@catholic.ac.kr; 2 Department of Laboratory Medicine, Incheon St. Mary’s Hospital, College of Medicine, The Catholic University of Korea, Seoul 06591, Republic of Korea; focused0922@gmail.com; 3 Department of Endocrinology, Bucheon St. Mary’s Hospital, College of Medicine, The Catholic University of Korea, Seoul 06591, Republic of Korea; sjyoomt@gmail.com; 4 Department of Clinical Medicine Research, Bucheon St. Mary’s Hospital, The Catholic University of Korea, Seoul 06591, Republic of Korea; kmmmmmy@naver.com; 5 Department of Laboratory Medicine, Seoul St. Mary’s Hospital, College of Medicine, The Catholic University of Korea, Seoul 06591, Republic of Korea

**Keywords:** microbiome, community state type, type 2 diabetes mellitus, SGLT2 inhibitor, candida

## Abstract

Type 2 diabetes mellitus (T2DM) significantly influences the composition and diversity of the vaginal microbiome, with implications for mucosal immunity, infection risk, and genitourinary health. This study aimed to investigate the vaginal microbiome profiles in women with T2DM, with a focus on differences according to menopausal status and associations with Candida colonization and the use of sodium–glucose cotransporter 2 (SGLT2) inhibitors. Compared to healthy controls, women with T2DM exhibited a decreased abundance of *Lactobacillus* species and increased microbial diversity. Community state of type (CST) IV, characterized by low *Lactobacillus* abundance and dominance of anaerobic taxa, was prevalent in the T2DM group. Among *Candida*-positive patients, *Lactobacillus iners*-dominant CST III was frequently observed, along with elevated levels of total and L-lactic acid. SGLT2 inhibitor users exhibited a different CST distribution pattern and slightly lower microbial richness and diversity, although these differences were not statistically significant. These findings underscore the impact of T2DM and its treatment on vaginal microbial composition and highlight the importance of considering vaginal health as part of comprehensive diabetes management in women.

## 1. Introduction

The composition of the vaginal microbiome undergoes dynamic changes throughout a woman’s life in response to both endogenous and exogenous factors, including age, menstruation, pregnancy, pharmacological treatments, urogenital infections [1], and underlying medical or anatomical conditions [2]. The vaginal microbiome plays a critical role in gynecological and reproductive health, with *Lactobacillus* species forming the primary line of defense against pathogenic infections [3]. Menopausal status is recognized as a major determinant of vaginal microbiome composition. Menopausal women exhibit a lower prevalence of *Lactobacillus*-dominant communities than premenopausal women, likely due to estrogen deficiency and the associated reduction in epithelial glycogen availability [4]. This hormonal shift facilitates a transition toward vaginal dysbiosis, commonly characterized by a predominance of community state of type (CST) IV, which is associated with a reduced *Lactobacillus* abundance and an increased susceptibility to genitourinary infections [5].

Vaginal CSTs represent a well-established framework for evaluating the compositional stability and functional state of the vaginal microbiome [6,7]. Temporal changes in CSTs have been documented across different stages of the female lifespan [4].

Type 2 DM (T2DM) is a chronic metabolic disorder associated with systemic complications such as retinopathy, neuropathy, and nephropathy [8], and is a strong independent predictor of genital infections [9]. Chronic hyperglycemia in T2DM leads to a sustained proinflammatory state, disrupting immune homeostasis and increasing susceptibility to infection [10,11]. In the vaginal environment, this manifests as an increased pH and altered microbial composition, further contributing to dysbiosis [4]. In addition to bacterial shifts, women with diabetes exhibit an elevated susceptibility to fungal infections, particularly *Candida* species [12,13,14]. *Lactobacillus iners-*dominant CSTs have been shown to harbor *Candida* more frequently than *L. crispatus-*dominant types, suggesting species-specific interactions between *Lactobacillus* and fungal colonization [15,16]. Although *L. iners* is frequently present in the vaginal microbiome, it is considered to provide suboptimal protection against *Candida* colonization [17] and may play an ambivalent role in maintaining vaginal health.

Pharmacological management of T2DM may further influence the vaginal microbiome. Sodium–glucose cotransporter 2 (SGLT2) inhibitors, which are increasingly prescribed for glycemic control, have been associated with a three-fold increased risk of genital infections, particularly during the early phase of treatment use [18,19]. Although the risk remains elevated during continued use, some studies suggest that the incidence of infections tends to decline with long-term administration [19]. Considering the high prevalence of vaginitis in women with T2DM and the complex interactions among microbial communities, mucosal immunity [4], and pharmacological interventions [20], elucidating these associations has important clinical implications.

A better understanding of these factors may enable early identification of individuals at risk for vaginal dysbiosis and inform individualized strategies for prevention and management, particularly in patients receiving SGLT2 inhibitors.

This study aimed to characterize the vaginal microbiome in women with T2DM, focusing on variations related to menopausal status, *Candida* infection, and SGLT2 inhibitor use. Additionally, vaginal cytokine profiles were analyzed to explore immune responses associated with microbial shifts and fungal infection.

## 2. Materials and Methods

### 2.1. Study Design and Subjects

All patients diagnosed with T2DM who visited our hospital between May 2020 and May 2022 were enrolled in the Department of Obstetrics and Gynecology Hospital. After obtaining informed consent, participants were screened to confirm their eligibility before enrollment. This prospective cohort study was conducted under the amended Declaration of Helsinki on the ethical conduct of research involving human subjects. The study was approved by the institutional scientific and ethical committees (protocol number: HC20TISIO034).

**Figure 1:**
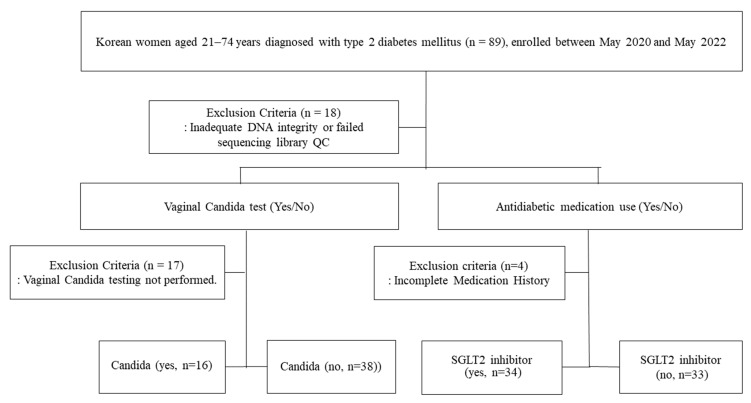
Participant enrollment, group stratification, and analysis workflow. For a comparison with healthy women (*n* = 100) [21], 16S rRNA gene sequencing data tagged as Project I from healthy, adult, North American females, collected from the posterior fornix and urogenital tracts, were obtained from the Public Database of the Human Microbiome Project, which was previously used in our previous study [22]. The vaginal microbiome taxonomic profile (MTP) correlations among the clinical data, Candida infection, medication (SGLT2 inhibitors), total lactic acid, L/D-lactic acid, glucose, and cytokines were analyzed depending on CST classification (*L. crispatus*-dominant CST group I, *L. gasseri*-dominant CST group II, *L. iners*-dominant CST group III, CST group IV with a low abundance of *Lactobacillus*, increased diversity and abundance of anaerobic species, CST group V with *L. jensenii*-dominant, and mixed CST with co-dominance of more than two *Lactobacillus* species).

Eighty-nine women aged 21 to 74 years who had been diagnosed with T2DM and had received medical treatment for at least six months were enrolled in the study. To minimize potential alterations in the vaginal microbiota, women who were using any topical vaginal treatments were excluded. Of these, 18 cases with DNA quality control (QC) and Library QC failures were excluded in the DNA extraction and 16S rRNA gene sequencing analyses. Finally, seventy-one women were enrolled in this study analysis (Figure 1).

### 2.2. Participant Data and Vaginal Sampling

Patient characteristics and medical histories were recorded. A standardized pelvic examination was performed using a sterile speculum in the lithotomy position. A physician collected participant samples from the posterior fornix of the vaginal wall, using a flocked swab (NFS-2 swab; Noble Biosciences, Inc., Whaseong-si, Republic of Korea) which was placed in 1 mL nucleic acid preservation media and 1 mL of phosphate-buffered saline. The collection of a vaginal swab sample in 1 mL nucleic acid preservation media and lysis steps were conducted at room temperature for 16 h, followed by centrifugation to remove particulate matter. The supernatants were then stored at −80 °C before DNA extraction processing. Another vaginal swab sample in 1 mL phosphate-buffered saline was stored at −80 °C before being assayed as described below.

Vaginal discharge was tested using PANA RealTyper™ STD Kit (HLB Panagene company, Daejeon, Republic of Korea), and the presence or absence of a Candida infection was determined.

### 2.3. Total Lactic Acid, L/D-Lactic Acid, Glucose Assays

To assess the metabolic activity of the vaginal microbiota and its association with CSTs, concentrations of total lactic acid, L- and D-lactic acid isomers, and glucose were measured. Lactic acid, predominantly produced by *Lactobacillus* species, plays a key role in maintaining vaginal homeostasis through its acidification and antimicrobial effects. The L/D-lactic acid ratio reflects species-specific metabolic pathways, while glucose serves as an essential metabolic substrate that may influence microbial composition, particularly in women with T2DM.

Vaginal swab samples stored in 1 mL of phosphate-buffered saline (PBS) were thawed on ice, vortexed for 10 s, and centrifuged at 2500 rpm for 10 min. The supernatant was transferred to a clean microcentrifuge tube. Total lactic acid, L- and D-lactic acid isomers, and glucose levels were quantified colorimetrically using EnzyChrom L-Lactate, D-Lactate, and Glucose Assay Kits (BioAssay Systems, Hayward, CA, USA), following the manufacturer’s instructions. For each assay, 20 μL of sample or standard was added to a clear-bottom 96-well plate. Absorbance was measured, and concentrations were calculated using standard curves generated in parallel. The lower limits of detection were 0.04 mM for L- and D-lactic acid and 0.05 μM for glucose.

### 2.4. Multiplex Immunoassay

To investigate differences in local immune profiles according to vaginal CSTs in women with T2DM, we measured the concentrations of ten cytokines in vaginal supernatants. Samples were analyzed for IFN-γ, IL-10, IL-12p70, IL-1β, IL-2, IL-4, IL-6, IL-8, MIP-1β, and TNF-α using the MILLIPLEX^®^ MAP Human Cytokine/Chemokine Magnetic Bead Panel (Millipore, Billerica, MA, USA), following the manufacturer’s instructions. Cytokine and chemokine levels were quantified using the FLEXMAP 3D system and analyzed with xPONENT^®^ software version 4.2 for the Luminex^®^ 200 platform (Luminex Corporation, Austin, TX, USA).

### 2.5. DNA Extraction and 16S rRNA Gene Sequencing

For DNA extraction, nucleic acid preservation media supernatants were thawed on ice. The 450 uL of supernatant was added to 300 of µL isopropanol and vortexed for 30 s. The mixture was placed in a PureLink™ Spin column from the PureLink™ Microbiome DNA purification kit (Thermo Fisher Scientific, Waltham, MA, USA) and DNA extraction was performed according to the manufacturer’s protocol. DNA concentration and purity were measured using a spectrophotometer (Eppendorf D30 (Eppendorf, Hamburg, Germany)). DNA concentration of more than or equal to 20 ng/μL with A260/A280 ratio of 1.8–2.0 was used for 16S rRNA sequencing. The genomic DNA was amplified by PCR, using fusion primers 341F (5′-AATGATACGGCGACCACCGAGATCTACAC-XXXXXXXX-TCGTCGGCAGCGTC-AGATGTGTATAAGAGACAG-CCTACGGGNGGCWGCAG-3′) and 805R (5′-CAAGCAGAAGACGGCATACGAGAT-XXXXXXXX-GTCTCGTGGGCTCGG-AGATGTGTATAAGAGACAG-GACTACHVGGGTATCTAATCC-3′; underlined sequences indicate the complementary regions of the primer) binding to the V3–V4 regions of the 16S rRNA gene, as previously reported [23].

### 2.6. Bioinformatics Analyses

The raw sequence reads were pre-processed for quality checks, including trimming of low-quality amplicons, diversity calculation, comparative MTP analysis, and taxonomic biomarker discovery analysis, using an EzBioCloud App (CJ Bioscience, Inc., Seoul, Republic of Korea), as previously reported [23]. Alpha diversity indices were calculated using ACE and Chao1 for species richness, and Shannon and NPShannon to measure species diversity and evenness, respectively. To visualize sample differences, beta diversity distances were calculated using principal coordinate analysis (PCoA) based on the Bray–Curtis method. A beta set-significance analysis between two MTP sets was performed using the permutational multivariate analysis of variance (PERMANOVA) test. Taxonomic biomarkers were displayed using the Linear Discriminant Analysis Effect Size-based cladogram [24].

### 2.7. Statistical Analyses

Statistical analyses were conducted using appropriate parametric or non-parametric methods, depending on data distribution. Normality was assessed prior to testing.

For two-group analyses, continuous variables were compared using either the unpaired *t*-test (for normally distributed data) or the Mann–Whitney U test (for non-normal distributions).

When comparing more than two groups, the one-way ANOVA was used for normally distributed data with equal variances, and the Kruskal–Wallis test was applied otherwise.

Fisher’s exact test was used to compare categorical variables, including CST IV versus non-CST IV distribution across clinical subgroups with limited sample sizes. D-/L-lactic acid concentrations, total lactic acid, and glucose levels were analyzed using the same methods based on data distribution.

A two-sided *p*-value < 0.05 was considered statistically significant. Analyses were performed using MedCalc version 22.016 (MedCalc Software, Ostend, Belgium), SPSS version 20.0 (IBM Corp., Armonk, NY, USA), GraphPad Prism 9.0 (GraphPad Software Inc., La Jolla, CA, USA), and Python version 3.11.8 with the SciPy library.

## 3. Results

### 3.1. Patient Demographics

A total of 71 Korean women with T2DM were included in the final analysis after the application of exclusion criteria (Figure 1). The clinical characteristics of the study participants are summarized in Table 1. All 71 participants were Korean women with T2DM, with a mean age of 49.3 ± 14.2 years, a mean body mass index (BMI) of 28.0 ± 5.4 kg/m^2^, and a mean HbA1c level of 8.2 ± 2.3%. Among the 54 participants who underwent Candida testing using the PANA RealTyper™ STD Kit, *Candida* infection was identified in 16 women (29.6%). Notably, 17 participants declined to undergo vaginal *Candida* testing. Compared to *Candida*-negative participants, those with *Candida*-positive results were significantly younger (*p* = 0.0015) and had a higher BMI (*p* = 0.0046). The *Candida*-positive group also included more premenopausal women (*p* = 0.006) and fewer patients with underlying medical conditions (*p* = 0.042). No significant differences were observed in HbA1c levels, gynecologic symptoms, or SGLT2 inhibitor use between the two groups.

Medication data were unavailable for 4 patients; therefore, 67 participants were included in the medication analysis. Among these, 45 patients (67.2%) were treated with oral antidiabetic agents alone, while 22 patients (32.8%) received combination therapy with insulin and oral medications (Table 2). Metformin was the most commonly prescribed oral agent, used in 60 patients (89.6%), followed by SGLT2 inhibitors in 34 (50.7%), dipeptidyl peptidase-4 (DPP-4) inhibitors in 7 (10.4%), and sulfonylureas in 4 (6.0%). Among patients treated with oral medications only, 18 of 19 SGLT2 inhibitor users (94.7%) were co-prescribed metformin. In the combination therapy group, 13 of the 15 patients (86.7%) receiving SGLT2 inhibitors were also on metformin.

### 3.2. Overall Sequence Output

Out of the 16S rRNA sequencing from patients’ samples (*n* = 71), the number of total valid reads ranged from 16,035 to 92,570 (median 33,065) after removing low quality amplicons, non-target amplicons, and chimeras. The number of OTUs ranged from 19 to 399 (median 55), and the number of species found in the samples ranged from 10 to 383 (median 40). The average percentage of valid reads identified at the species level was 99.64 ± 0.77% (mean ± SD).

### 3.3. Alterations in Vaginal Community State Types in Women with T2DM: Associations with Menopausal Status, Candida Infection, and SGLT2 Inhibitor Use

**Figure 2:**
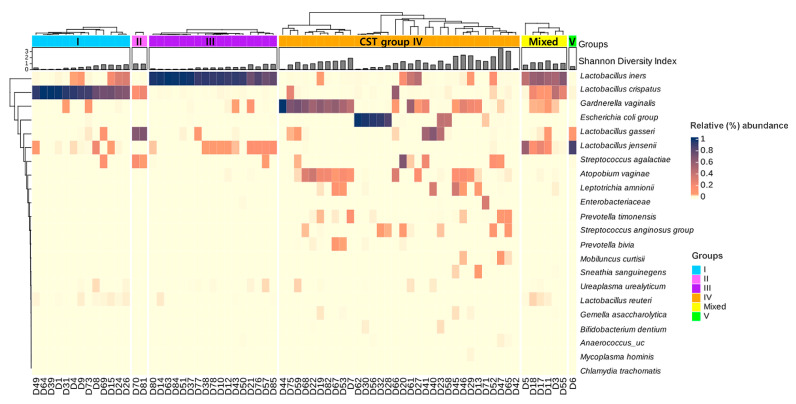
Heatmap showing the relative abundances of 22 representative microbial taxa at the species level in the vaginal microbiome of 71 women with T2DM. Hierarchical clustering revealed distinct grouping patterns according to CSTs, primarily defined by the dominant *Lactobacillus* species. The color scale indicates the relative abundance (%) of each taxon.

A heatmap analysis was performed to assess the relative abundances (%) of 22 representative vaginal microbial species in the 71 women with T2DM (Figure 2). Hierarchical clustering identified distinct CST profiles, defined by dominant *Lactobacillus* species. CST IV and Mixed groups exhibited greater microbial diversity and higher proportions of anaerobic bacteria. These CSTs were more frequently observed in women with T2DM, whereas CST I appeared at a lower frequency overall but was still detected in several subgroups.

**Figure 3:**
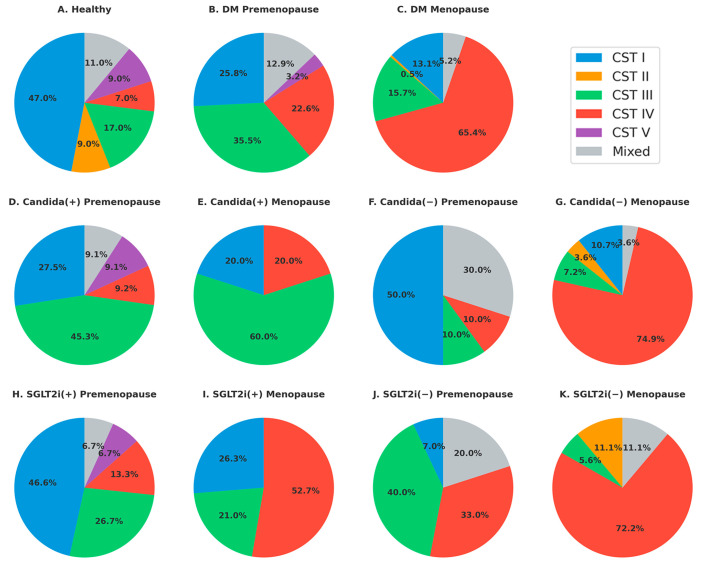
Representation of vaginal bacterial CSTs across clinical subgroups of women. (**A**) Healthy women (*n* = 100); (**B**) women with T2DM, premenopausal; (**C**) T2DM, menopausal; (**D**) T2DM with *Candida* infection, premenopausal; (**E**) T2DM with *Candida* infection, menopausal; (**F**) T2DM without *Candida* infection, premenopausal; (**G**) T2DM without *Candida* infection, menopausal; (**H**) T2DM with SGLT2 inhibitor use, premenopausal; (**I**) T2DM with SGLT2 inhibitor use, menopausal; (**J**) T2DM without SGLT2 inhibitor use, premenopausal; (**K**) T2DM without SGLT2 inhibitor use, menopausal. CSTs: CST I (blue, *Lactobacillus crispatus*-dominant), CST II (orange, *Lactobacillus gasseri*-dominant), CST III (green, *Lactobacillus iners*-dominant), CST IV (red, *Lactobacillus*-depleted, anaerobe-rich), CST V (purple, *Lactobacillus jensenii*-dominant), Mixed (gray, heterogeneous or non-dominant composition).

The distribution of vaginal CSTs varied substantially across clinical subgroups (Figure 3). In healthy women (A), CST I was the most predominant (47%), followed by CST III (17%) and CST II (9%), consistent with a typical *Lactobacillus*-dominant vaginal microbiota.

In women with T2DM, a shift toward CST III and CST IV was observed, especially among menopausal women. The prevalence of CST I decreased from 25.8% in premenopausal women (B) to 13.1% in menopausal women (C), while CST IV increased from 22.6% to 65.4% (*p* = 0.002, Fisher’s exact test), suggesting a menopause-related disruption in *Lactobacillus* stability and increased dysbiosis.

Among women with T2DM with a Candida infection (D, E), CST III was the predominant community type (45–60%). In Candida-negative cases (F, G), CST IV was most prevalent in menopausal women (G: 74.9%). In contrast, CST I was relatively preserved in premenopausal Candida-negative women (F: 50%), who also exhibited the highest proportion of mixed CSTs (30%). However, Candida infection status was not significantly associated with the distribution of CST IV among menopausal women (*p* = 1.000, Fisher’s exact test).

In premenopausal women, SGLT2 inhibitor users (H) exhibited a higher proportion of CST I (46.6%) and a lower proportion of CST IV (13.3%). In contrast, among non-users (J), CST IV accounted for 33.0%, making it the second most common CST following CST III (40.0%).

In menopausal women, CST IV remained the predominant community type in both SGLT2 inhibitor users (I) and non-users (K); however, its prevalence was lower in users (52.7%) than in non-users (72.2%). CST I was detected in 26.3% of SGLT2 inhibitor users (I) but was not observed in non-users (K). When both premenopausal and menopausal women were considered together, CST IV was less prevalent in SGLT2 inhibitor users (H and I) than in non-users (J and K), although this difference did not reach statistical significance (*p* = 0.079, Fisher’s exact test).

**Figure 4:**
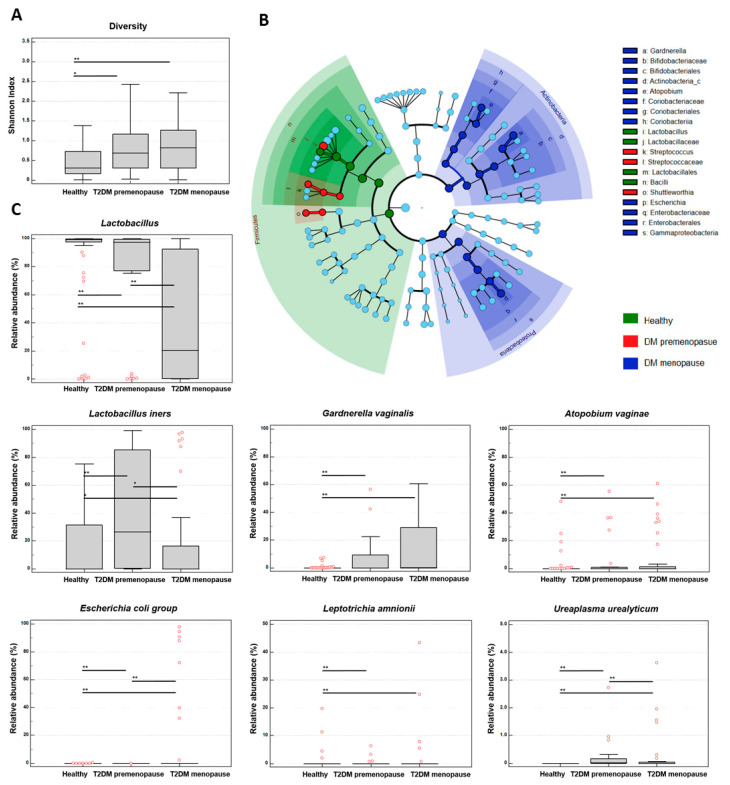
Differences in vaginal MTPs among healthy women and women with T2DM, stratified by menopausal status. (**A**) Shannon diversity index was significantly higher in both the premenopausal (*n* = 31) and menopausal women with T2DM (*n* = 40) groups compared to healthy women (*n* = 100) (*p* = 0.0006, Mann–Whitney U test). (**B**) Cladogram based on LEfSe analysis showing phylogenetic distribution of significantly enriched taxonomic biomarkers (linear discriminant analysis [LDA] score ≥ 4) among healthy, T2DM premenopausal, and T2DM menopausal groups. (**C**) Relative abundances (%) of significantly different vaginal microbial taxa among healthy women, premenopausal women with T2DM, and menopausal women with T2DM. Red circles indicate individual sample values. Statistical significance was determined using non-parametric tests; *p* < 0.05 (*), *p* < 0.01 (**).

The Shannon diversity index was significantly higher in premenopausal and menopausal women with T2DM compared to healthy women (median 0.696 vs. 0.824 vs. 0.304, respectively; *p* = 0.0023, Kruskal–Wallis test) (Figure 4A). No significant differences were observed in the Chao1 richness index among the three groups (median 28.250 vs. 42.167 vs. 29.925; *p* = 0.052).

The beta diversity distances also showed statistically significant differences across groups (*p* = 0.0001, PERMANOVA, 999 permutations).

The LEfSe analysis-based cladogram illustrated the phylogenetic distribution of significantly enriched taxonomic biomarkers (LDA score ≥ 4) among premenopausal women with T2DM, menopausal women with T2DM, and healthy women (Figure 4B). Specifically, the phylum *Actinobacteria* and its subordinate taxa, including the genera *Atopobium*, *Gardnerella*, and *Escherichia*, were most enriched in menopausal women with T2DM. The genera *Streptococcus* and *Shuttleworthia* were predominant in the T2DM premenopausal group, while the phylum *Firmicutes* and the genus *Lactobacillus* were most enriched in healthy women.

We compared the relative abundances of vaginal microbial taxa among the three groups (Figure 4C). The genus *Lactobacillus* and its species (*L. crispatus*, *L. gasseri*, *L. jensenii*, *L. reuteri*) showed significantly higher relative abundances in healthy women (*p* < 0.01). In contrast, the relative abundances of *L. iners*, *Gardnerella vaginalis*, *Atopobium vaginae*, *Escherichia coli* group, *Leptotrichia amnionii*, and *Ureaplasma urealyticum* were significantly higher in both premenopausal and menopausal women with T2DM (*p* < 0.05 or *p* < 0.01, non-parametric test).

### 3.4. Comparison of Vaginal Microbial Taxonomic Profiles in T2DM Patients with and Without Candida Infection

**Figure 5:**
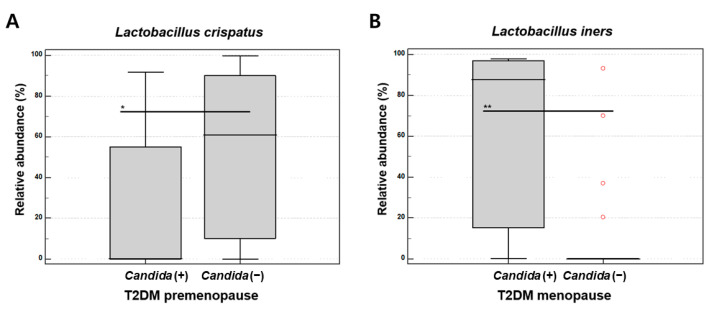
Relative abundance of *Lactobacillus* species by Candida infection status in women with T2DM. (**A**) Decreased *L. crispatus* in Candida-positive premenopausal women. (**B**) Increased *L. iners* in Candida-positive menopausal women. Non-parametric test; *p* < 0.05 (*), *p* < 0.01 (**).

Among premenopausal women with T2DM, there were no statistically significant differences in species richness (Chao1, *p* = 0.205) or diversity (Shannon, *p* = 0.778) between those with and without *Candida* infection. Beta diversity also showed no significant difference (PERMANOVA, *p* = 0.08). However, the relative abundance of *Lactobacillus crispatus* was significantly lower in the *Candida*-positive group (median 0.380% vs. 60.783%, *p* = 0.041, in non-parametric test) (Figure 5A).

In menopausal women with T2DM, species richness (Chao1, *p* = 0.315) and diversity (Shannon, *p* = 0.145) did not differ significantly between groups. However, the beta diversity differed significantly (PERMANOVA, *p* = 0.014). The relative abundance of *Lactobacillus iners* was significantly higher in women with *Candida* infection (median 87.639% vs. 0.058%, *p* = 0.0022, in non-parametric test) (Figure 5B).

### 3.5. No Stastically Different Vaginal MTP in T2DM Premenopause and T2DM Menopause According to Taking SGLT2 Inhibitors

The alpha diversity indices of species richness (*p* = 0.120 for Chao1) and diversity (*p* = 0.351 for Shannon) showed no statistically significant differences between premenopausal women with T2DM taking SGLT2 inhibitors (*n* = 15) and those not taking SGLT2 inhibitors (*n* = 15). The beta diversity distance was also not statistically significant differences between the two groups (*p* = 0.08 by PERMANOVA with 999 permutations).

Likewise, the alpha diversity indices of species richness (*p* = 0.213 for Chao1) and diversity (*p* = 0.089 for Shannon) showed no statistically significant differences between menopausal women with T2DM taking SGLT2 inhibitors (*n* = 19) and those not taking SGLT2 inhibitors (*n* = 18).; the beta diversity distance showed no statistically significant differences between the two groups (*p* = 0.384 by PERMANOVA with 999 permutations).

### 3.6. L/D-Lactic Acid, Glucose, and Cytokine Profiles by CST and Candida Infection Status

**Figure 6:**
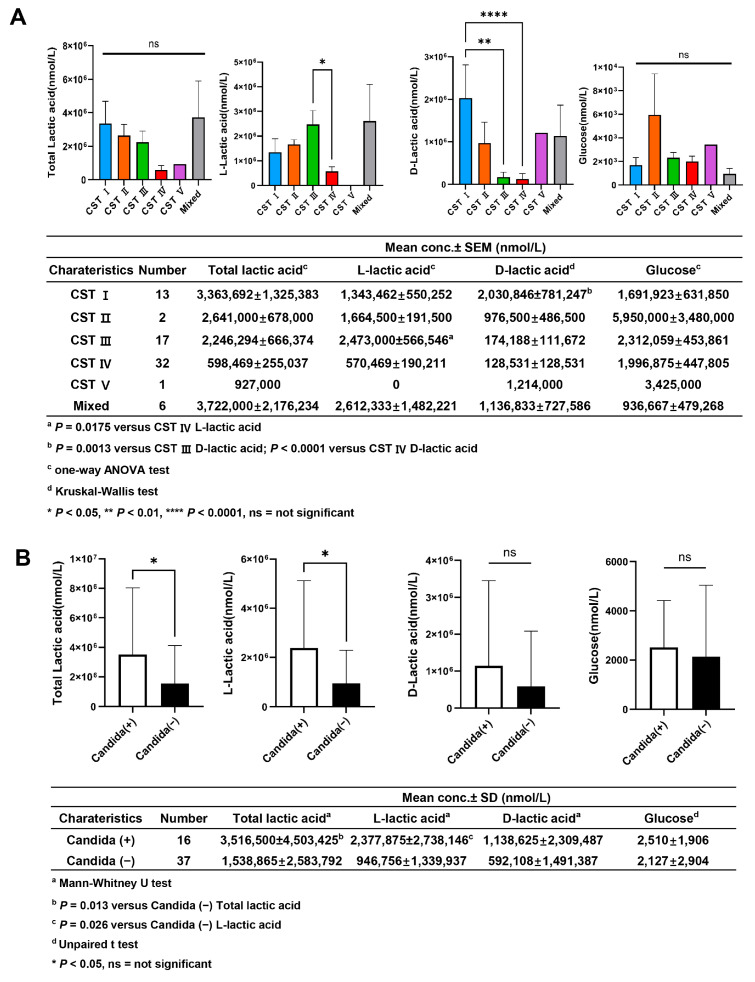
Vaginal lactic acid and glucose concentrations in women with T2DM, according to CST and *Candida* infection status. (**A**) L-lactic acid highest in CST III; D-lactic acid highest in CST I (*p* < 0.05). No difference in total lactic acid or glucose. (**B**) Higher total and L-lactic acid in Candida-positive vs. Candida-negative (*p* = 0.013, 0.026). No difference in D-lactic acid or glucose. Non-parametric test; *p* < 0.05 considered significant.

L- and D-lactic acid concentrations varied by CST classification, with significantly higher L-lactic acid levels observed in CST III and higher D-lactic acid levels in CST I (*p* < 0.05 for both). No significant differences were found in total lactic acid or glucose levels across the CST groups (Figure 6A). When stratified by *Candida* infection status, total lactic acid and L-lactic acid concentrations were significantly higher in women with a *Candida* infection compared to those without an infection (*p* = 0.013 and *p* = 0.026, respectively), whereas D-lactic acid and glucose levels did not differ significantly between the groups (Figure 6B). Menopausal status did not significantly influence total lactate, D-/L-lactate, or glucose concentrations across CST groups or according to Candida infection status.

CST IV was associated with higher levels of proinflammatory cytokines, including IL-1β, IL-12p70, and TNF-α, as well as the anti-inflammatory cytokine IL-10, compared to CST I (Figure S1)

## 4. Discussion

This study comprehensively investigated alterations in the vaginal microbiome among women with T2DM, focusing on the influence of menopausal status, *Candida* infection, and the administration of SGLT2 inhibitors. In addition, we examined the associations between microbial community composition and local metabolic factors, including lactic acid and glucose concentrations, as well as inflammatory cytokine profiles. These findings contribute to a better understanding of the interplay between host metabolism, vaginal microbial composition, and mucosal immunity in women with diabetes, and may help inform future approaches to the prevention and management of genital tract inflammation in this population.

The T2DM patients exhibited greater microbial heterogeneity than the healthy women, as indicated by increased Shannon diversity indices despite a comparable species richness. Beta diversity analyses revealed distinct clustering patterns, reflecting a compositional shift associated with the diabetic state. The taxonomic analyses demonstrated an enrichment of *Actinobacteria* taxa, including *Atopobium, Gardnerella*, and *Escherichia*, particularly in menopausal women. In contrast, *Firmicutes* and *Lactobacillus* were more prevalent in healthy women, indicating a more stable and protective microbiota.

In healthy women, CST I and CST III were the dominant community state types, reflecting a *Lactobacillus-*rich vaginal microbiota [7]. CST III has also been frequently reported among healthy Asian populations [7]. Women with T2DM, especially those who were menopausal, exhibited a marked reduction in CST I and a shift toward CST III and CST IV. This microbial shift may reflect the destabilization of *Lactobacillus*-dominant communities during the menopausal transition. CST IV, characterized by a higher abundance of *Gardnerella* and *Prevotella*, is commonly associated with vaginal dysbiosis and has been reported in studies of recurrent vaginitis [23] and pelvic organ prolapse [2].

*Candida* vulvovaginitis was observed in 29.6% of women tested and was associated with younger age, higher BMI, and premenopausal status. CST III predominated in *Candida-*positive individuals, while CST IV was more frequent in *Candida*-negative women, although the association was not statistically significant. These patterns suggest that *Candida* infection is modulated by complex host and environmental factors. At the species level, *L. iners* was dominant in women with a *Candida* infection, often replacing *L. crispatus*, a key species in maintaining a low vaginal pH. *L. iners*, which produces only L-lactic acid, was commonly found alongside *Gardnerella* and *Prevotella*, reinforcing its association with dysbiosis [1,25,26].

*Candida* infection was not associated with significant changes in overall microbial diversity, but species-level shifts in *Lactobacillus* composition were evident. Specifically, *L. crispatus* decreased while *L. iners* increased in *Candida*-positive individuals. This suggests that an imbalance in *Lactobacillus* species, rather than broad community disruption, may underlie *Candida* infection in T2DM patients.

SGLT2 inhibitor use appeared to influence vaginal microbiome composition [20]. Among premenopausal women, SGLT2 inhibitor users showed higher prevalence of CST I and III and lower CST IV, indicating a potential protective role in maintaining *Lactobacillus*-dominant communities, although these differences were not statistically significant. In menopausal women, CST IV predominated regardless of SGLT2 inhibitor use, although CST I was observed only in users. These trends warrant further investigation into the microbial impact of SGLT2 inhibitors.

SGLT2 inhibitor users showed lower species richness and alpha diversity than non-users, but the differences were not significant. Candida prevalence was similar between groups. These findings should be interpreted in the context of host factors and concomitant treatments.

Metformin was the most frequently prescribed antidiabetic agent and was commonly co-administered with SGLT2 inhibitors, reflecting standard treatment practices. The potential confounding effects of pharmacologic co-exposures must therefore be considered in microbiome analyses. These observations highlight the multifactorial nature of vaginal microbiome composition in T2DM, shaped by metabolic, hormonal, and pharmacologic influences. *L. iners* was consistently detected across both eubiotic and dysbiotic states [1,25,27,28,29], and its frequent co-occurrence with *Candida* and anaerobes underscores the need for mechanistic studies to clarify its ecological role.

Vaginal metabolite profiles varied according to CST and *Candida* infection status. Lactic acid, essential for vaginal pH and immune balance, exists in two isomeric forms. L-lactic acid was significantly elevated in CST III (*L. iners* dominant), while D-lactic acid was highest in CST I (*L. crispatus* dominant) [30,31]. These patterns align with known metabolic traits of these species [30]. Stratification by *Candida* infection revealed that total and L-lactic acid concentrations were significantly higher in *Candida-*positive women (*p* = 0.013 and *p* = 0.026, respectively), whereas D-lactic acid and glucose levels did not differ significantly. This indicates that shifts in lactic acid isomer profiles particularly increased L-lactic acid may be more influential than hyperglycemia in shaping the vaginal environment during *Candida* infection. Elevated lactic acid may reflect microbial competition or adaptive metabolic responses in dysbiotic states [31,32].

Rodrigues et al. [12] previously reported that elevated HbA1c was associated with vulvovaginal candidiasis in diabetic patients. In contrast, our study found no significant differences in HbA1c or vaginal glucose concentrations between *Candida*-positive and -negative groups. This discrepancy may be attributed to uniformly stable glycemic control in our cohort under antidiabetic treatment. Although a direct association between hyperglycemia and the presence of *Candida* was not observed, a complex interplay among host factors, microbial communities, and metabolite dynamics is likely present in women with T2DM.

In this study, menopausal status did not significantly affect vaginal levels of lactic acid or glucose across CST groups or by *Candida* status. Alterations in microbial composition, including shifts in dominant *Lactobacillus* species, may play a key role in shaping the vaginal metabolic environment. In women with T2DM, disruption of the vaginal glucose–lactic acid axis may impair microbial homeostasis and increase susceptibility to infection and inflammation. Chronic hyperglycemia may elevate mucosal glucose availability, thereby promoting the growth of opportunistic pathogens while suppressing lactic acid production by *Lactobacillus* species [25,31]. Notably, *Lactobacillus iners* was consistently detected in both eubiotic and dysbiotic states and frequently co-occurred with *Candida* and anaerobic taxa, underscoring its context-dependent ecological role and supporting the need for further mechanistic investigation [1,27,28,29]. These findings suggest that vaginal metabolic alterations in women with T2DM may be primarily influenced by microbial community structure and infection status. Although menopausal hormonal changes did not show a direct effect in this study, their potential contribution cannot be excluded and warrants further investigation.

CST IV was more frequently observed in women with T2DM than in healthy controls and was associated with higher levels of IL-1β, IL-12p70, TNF-α, and IL-10. This cytokine profile suggests alterations in the local mucosal immune environment that may be related to changes in microbial composition. The balance between proinflammatory and anti-inflammatory cytokines in the vagina is thought to be influenced by the dominant microbial taxa. Conversely, shifts in cytokine levels may signal the activation of local immune responses, including both cellular and humoral components, which could in turn modulate the microbial community. Cytokine profiling may therefore provide useful insights into host–microbiome interactions in T2DM.

These findings are consistent with reports showing that CST IV—characterized by low levels of *Lactobacillus* and enriched anaerobes—is linked to inflammation and immune activation [19,33]. In addition, reduced lactic acid and *Lactobacillus* depletion may impair mucosal defense and foster a proinflammatory environment conducive to dysbiosis and opportunistic infection [32].

This study has several limitations. First, all participants had been diagnosed with T2DM for over six months, limiting insights into early microbiome changes after diagnosis. Second, many participants received combination therapy (typically metformin plus SGLT2 inhibitors), complicating the attribution of microbial effects to individual agents. Third, approximately one-quarter of participants were not tested for vaginal *Candida*, introducing a potential misclassification bias. Fourth, a lack of baseline microbiome data from healthy Korean women limits population specific interpretations.

## 5. Conclusions

This study demonstrates that women with T2DM exhibit an increased vaginal microbial diversity and reduced *Lactobacillus* dominance, particularly after menopause. *Candida* infection was associated with CST III dominated by *Lactobacillus iners*, while SGLT2 inhibitor use in premenopausal women was linked to more favorable CST distributions. These findings suggest the importance of microbiome-based strategies for preventing dysbiosis and inflammation in women with T2DM.

## Figures and Tables

**Table 1:** Baseline clinical characteristics of women with T2DM, stratified by *Candida* colonization status and SGLT2 inhibitor use.

Characteristics	Total	Candida (+)	Candida (−)	*p* Value	SGLT2 Inhibitor (+)	SGLT2 Inhibitor (−)	*p* Value
Number	71	16	38		34	33	
Age (years)	49.3 ± 14.2	42.8 ± 11.3	55.5 ± 12.6	0.0015 ^a^	48.9 ± 14.4	49.4 ± 13.9	0.891 ^a^
Height (cm)	158 ± 6.6	161.4 ± 3.4	156.1 ± 7.3	0.0095 ^a^	158 ± 6.8	158.4 ± 6.6	0.820 ^a^
Weight (kg)	70.5 ± 17.3	80.6 ± 12.2	66.3 ± 10.7	0.0001 ^a^	71.8 ± 12.6	69.8 ± 21.4	0.654 ^a^
BMI (Kg/m^2^)	28 ± 5.4	30.8 ± 4.4	27.2 ± 3.8	0.0046 ^a^	28.8 ± 4.5	27.4 ± 6.2	0.304 ^a^
Menopause				0.006 ^b^			1.000 ^b^
Premenopausal (%)	31 (43.7%)	11 (68.6%)	10 (26.3%)		15 (44.1%)	15 (45.5%)	
Menopausal (%)	40 (56.3%)	5 (31.3%)	28 (73.7%)		19 (55.9%)	18 (54.5%)	
Mean HbA1C (%)	8.2 ± 2.3	8.3 ± 1.7	7.9 ± 2.2	0.6422 ^a^	8.6 ± 2.3	7.6 ± 2.2	0.0057 ^c^
Gynecologic symptom (vaginal discharge, itching, odor, pain)				0.141 ^b^			0.447 ^b^
no	23 (34.3%)	1 (6.3%)	10 (27%)		10 (29.4%)	13 (39.4%)	
yes	44 (65.7%)	15 (93.8%)	27 (73%)		24 (70.6%)	20 (60.6%)	
Underlying medical diseases (HTN, CVA Thyroid disease)				0.042 ^b^			0.745 ^b^
no	43 (60.6%)	11 (68.6%)	20 (52.6%)		20(58.5%)	20 (60.6%)	
yes	28 (39.4%)	5 (31.4%)	18 (47.4%)		14 (41.2%)	13 (39.4%)	
Urological symptoms (frequency, urgency %)				1.000 ^b^			0.259 ^b^
no	60 (84.5%)	12 (87.5%)	31 (81.6%)		28 (82.4%)	31 (93.9%)	
yes	11 (15.5%)	4 (12.5%)	7 (18.4%)		6 (17.6%)	2 (6.1%)	

^a^ Unpaired *t*-test; ^b^ Fisher’s exact test; ^c^ Mann–Whitney U test. Data for continuous variables are reported as mean ± SD. A total of 18 participants were excluded from the analyses due to the absence of vaginal Candida testing, and 4 participants were excluded because of incomplete documentation of their medication history.

**Table 2:** Classification of antidiabetic medication use among study participants.

	Oral Medications Only (*n* = 45)	Insulin + Oral Medications, (*n* = 22)
Metformin(+)/SGLT2 inhibitor	18	13
Metformin(+)/Others	23	6
Metformin(−)/SGLT2 inhibitor	1	2
Metformin(−)/Others	3	1

## Data Availability

The 16S rRNA gene sequences have been submitted to the NCBI Sequence Read Archive SRA and are available under the BioProject ID PRJEB73978.

## Supplementary Materials

The following supporting information can be downloaded at: https://www.mdpi.com/article/10.3390/microorganisms13061426/s1, Figure S1: Comparison of vaginal cytokine profiles across CST groups.

## Abbreviations

The following abbreviations are used in this manuscript:

BMI	Body Mass Index
CST	Community Status Type
DM	Diabetes Mellitus
MTP	Microbiome Taxonomic Profile
SGLT2	Sodium–Glucose Cotransporter 2
PBS	Phosphate-Buffered Saline
PCoA	Principal Coordinate Analysis
T2DM	Type 2 Diabetes Mellitus
QC	Quality Control
