# Supplementary material for: The Clinically Significant Changes in the Composition and Functional Diversity of the Vaginal Microbiome in Women with Type 2 Diabetes Mellitus
Source: Microorganisms. 2025 Jun 19;13(6):1426. doi: 10.3390/microorganisms13061426 (PMC12195554; doi:10.3390/microorganisms13061426)
Supplement: Supplementary file 1 [file microorganisms-13-01426-s001.zip › microorganisms-3586774-supplementary.pdf]

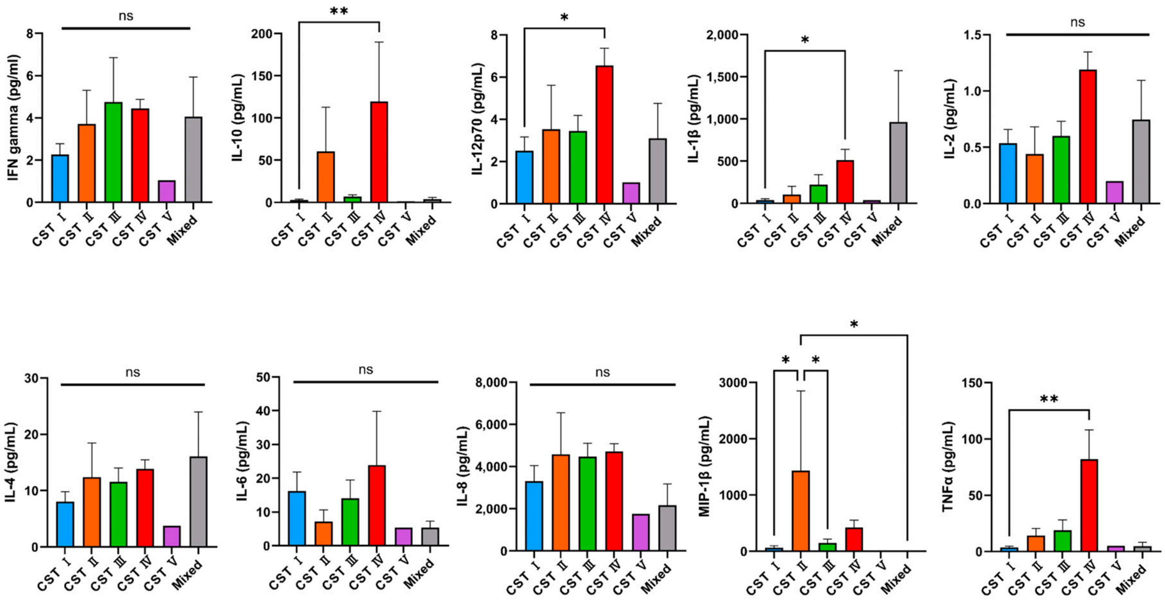

		Mean conc.± SEM (pg/mL)									
Charateristics	Number	IFNγ[h]	IL-10[i]	IL-12P70[h]	IL-1β[i]	IL-2[h]	IL-4[h]	IL-6[h]	IL-8[h]	MIP-1β[h]	TNFα[i]
CST Ⅰ	13	2.27±0.50	2.99±7.38[a]	2.50±0.65[b]	39.63±16.03[c]	0.53±0.13	8.05±1.80	16.19±5.61	3309.49±726.89	64.23±34.44[d]	3.55±1.18[g]
CST Ⅱ	2	3.72±1.6	60.25±52.54	3.53±2.10	104.75±96.15	0.44±0.24	12.39±6.1	7.18±3.41	4580.50±1966.50	1434.07±1415.94[e,f]	14.14±6.27
CST Ⅲ	17	4.75±2.11	6.89±2.27	3.44±0.73	224.00±115.41	0.60±0.13	11.57±2.46	14.10±5.38	4470.68±629.34	145.33±70.35	19.07±9.03
CST Ⅳ	32	4.45±0.43	119.52±70.41	6.55±0.82	510.31±131.32	1.20±0.15	13.88±1.59	23.95±15.82	4718.01±364.70	427.12±127.69	82.06±25.91
CST Ⅴ	1	1	1.45	1.01	37.38	0.20	3.77	5.43	1765	15.92	5.04
Mixed	6	4.05±1.88	3.91±2.12	3.09±1.66	963.55±609.51	0.75±0.35	16.10±7.87	5.40±1.86	2167.42±1011.87	6.77±1.47	4.71±3.33

[a] *P* = 0.0038 versus CST Ⅳ IL-10
[b] *P* = 0.0258 versus CST Ⅳ IL-12p70
[c] *P* = 0.0226 versus CST Ⅳ IL-1β
[d] *P* = 0.0309 versus CST Ⅱ MIP-1β
[e] *P* = 0.0444 versus CST Ⅲ MIP-1β
[f] *P* = 0.0398 versus Mixed MIP-1β
[g] *P* = 0.0014 versus CST Ⅳ TNFα
[H] one-way ANOVA test
[i] Kruskal-Wallis test
* $P < 0.05$, ** $P < 0.01$, ns = not significant

Figure S1. Comparison of vaginal cytokine profiles across CST groups.
